# The biologic era and pregnancy in rheumatoid arthritis: a prospective multicenter study from the Norwegian RevNatus registry

**DOI:** 10.1186/s41927-026-00663-7

**Published:** 2026-06-03

**Authors:** Ingunn Sagberg, Carina Gøtestam Skorpen, Stian Lydersen, Ingvild Birgitte Refsnæs, Anna-Birgitte Aga, Bente Jakobsen, Hege Suorza Svean Koksvik, Marianne Wallenius

**Affiliations:** 1 Norwegian Research Center for Women’s Health, Oslo University Hospital, Oslo, Norway; 2 Institute of Neuromedicine and Movement Science, Norwegian University of Science and Technology (NTNU), Trondheim, Norway; 3 Norwegian National Network for Pregnancy and Rheumatic Diseases, Department of Rheumatology, St. Olavs Hospital Trondheim University Hospital, Trondheim, Norway; 4 Department of Health Sciences, Faculty of Medicine and Health Sciences, Norwegian University of Science and Technology (NTNU), Ålesund, Norway; 5 Department of Rheumatology, Ålesund Hospital, Ålesund, Norway; 6 Department of Mental Health, Regional Centre for Child and Youth Mental Health and Child Welfare, Faculty of Medicine and Health Science, Norwegian University of Science and Technology (NTNU), Trondheim, Norway; 7 Department of Rheumatology, Oslo University Hospital, Oslo, Norway; 8 Diakonhjemmet Hospital, REMEDY Center for Treatment of Rheumatic and Musculoskeletal Diseases, Oslo, Norway

**Keywords:** Rheumatoid arthritis, Pregnancy, Amelioration, Postpartum, Flare, bDmards

## Abstract

**Background:**

It is known that pregnancy affects disease activity in Rheumatoid arthritis (RA), but earlier studies have shown conflicting results regarding the degree of change and the role of antibodies. Few studies have been conducted on patients with use of Tumor Necrosis Factor-α Inhibitors (TNFi) throughout pregnancy. The aims of this study were to investigate disease activity in women with RA from preconception to 12 months postpartum and assess the influence of anti-cyclic citrullinated protein antibodies (ACPA), rheumatoid factor (RF), erosive disease, and time trends.

**Methods:**

This prospective multicenter cohort study used data from the Norwegian RevNatus registry (2007–2024). Women with RA were followed before pregnancy, each trimester, and until 12 months postpartum. We assessed disease activity using Disease Activity Score in 28 joints with C-Reactive Protein (DAS28(3)CRP). Linear mixed models were used to evaluate changes over time and interactions with ACPA, RF and erosions, and we divided pregnancies into three groups by year of delivery. Flares were defined as a change of DAS28(3)CRP > 1.2.

**Results:**

A total of 598 pregnancies in 475 women were included. The estimated mean DAS28(3)CRP decreased from 2.39 in 2^nd^ to 2.27 in 3^rd^ trimester (*p* = 0.003) and increased significantly to 2.52 (*p* < 0.001) at 6 weeks postpartum with 16% experiencing flares. The proportion improving during pregnancy was 11%, but 49% in those with DAS28(3)CRP > 3.2 in 1^st^ trimester. Seropositive women had more fluctuations during pregnancy and higher postpartum activity; seronegative women showed minimal variation. Erosive disease did not have a significant effect. Disease activity before, during and after pregnancy decreased in later years of delivery, corresponding to increased use of TNFi.

**Conclusion:**

Most women with RA maintained remission during pregnancy, but postpartum flares still occurred. Improvement was seen in pregnancies with initial high disease activity, but not in women with well managed disease. Seropositive women experienced higher disease activity and larger increase in DAS28(3)CRP postpartum. Women with pregnancies after 2020 had consistently lower disease activity compared to those who were pregnant in 2007–2019, coinciding with broader TNFi use during pregnancy.

**Supplementary Information:**

The online version contains supplementary material available at 10.1186/s41927-026-00663-7.

## Key message

**What is already known on this topic**

• Pregnancy affects disease activity in RA, but earlier studies have shown conflicting results regarding the degree of change and the role of antibodies.

• The treatment of RA in pregnancy has changed in the last 10 years with increased use of tumor necrosis factor inhibitors (TNFi). Few studies have been conducted on patients with TNFi-use throughout pregnancy.

**What this study adds**

• The largest study investigating disease activity both before and after the introduction of TNFi in pregnancy.

• This study showed improvement in pregnancies with initial high disease activity, but not in the women with well managed disease.

• Women with RA who were pregnant after 2020 had consistently lower disease activity compared to those who were pregnant in 2007-2019, coinciding with broader TNFi use during pregnancy.

**How this study might affect research, practice, or policy**

• This study adds to the knowledge of what to expect when becoming pregnant for women with RA and underscores the importance of personalized advice and treatment.

## Background

Rheumatoid arthritis (RA) is a systemic autoimmune disease that causes inflammation in joints and may affect internal organs. The disease often affects women of fertile age, and rheumatologists have long studied the relationship between RA and pregnancy. The earliest reports described a near-complete remission in almost all women during pregnancy [1]. Later studies with a larger number of patients and more objective measures of disease activity did not find the same unambiguous results regarding improvement. A meta-analysis in 2019 found that 60% of patients with RA improved during pregnancy and 47% relapsed postpartum [2]. Later studies with a higher percentage of patients using biological disease-modifying anti-rheumatic drugs (bDMARDs) have not found any significant differences in disease activity during pregnancy [3, 4]. Earlier studies have also found conflicting results regarding the presence of anti-cyclic citrullinated peptide (ACPA), Rheumatoid Factor (RF), and erosions and their possible associations with disease activity during pregnancy [5, 6].

The introduction of bDMARDs has transformed the treatment of RA. The first tumor necrosis factor-α inhibitor (TNFi) was introduced in 1998, but it was initially not considered safe for use in pregnancy [7]. The use has increased in the last decade after The European Alliance of Associations for Rheumatology (EULAR) published their first guidelines for the use of bDMARDs in pregnancy in 2016 [8] and The American College of Rheumatology (ACR) in 2020 [9], both supporting increased use of TNFi during pregnancy [7, 10]. The optimal treatment length of bDMARDs during pregnancy is not yet established.

The objectives of this study were to investigate disease activity in women with RA from preconception, throughout pregnancy, and one year postpartum. Next, to assess whether the presence of ACPA, RF, or erosive disease was associated with increased disease activity, and finally, to explore how disease activity in pregnancy has changed over time.

## Methods

### Study design

This prospective multicenter study included patients from the nationwide Norwegian registry on pregnancy and rheumatic diseases, RevNatus. The register was established in 2006, now including 19 centers, and is managed by the Norwegian National Network for Pregnancy and Rheumatic Diseases (NKSR) [11]. Female patients with a rheumatic disease, 16 years or older, qualify for inclusion before or during pregnancy. From the time of inclusion, patients are followed throughout pregnancy at their local rheumatology unit with one visit each trimester and at six weeks, six months, and 12 months after birth.

### Study population

**Fig. 1:**
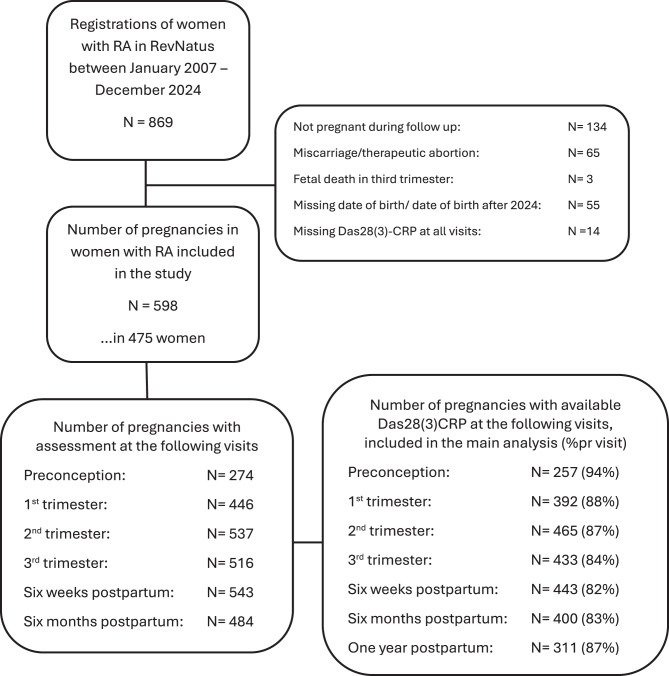
Flowchart of pregnancies in women with rheumatoid arthritis included in the study, and numbers of pregnancies at each study time point. RA, rheumatoid Arthritis; DAS28(3)CRP, disease activity score with 28 joints with C-reactive protein

Included in the study were women with RA registered in RevNatus in the period January 2007 to December 31st, 2024. All patients met the ACR 1987 or ACR/EULAR 2010 classification criteria for RA. Exclusion criteria are listed in Fig. 1. We included women with at least one visit with registered DAS28(3)CRP during the follow-up period.

### Data collection

Data collection in RevNatus is ideally at seven time points, however, not all women attend all visits for various reasons. At inclusion demographic data, nicotine use, comorbidities, information regarding earlier pregnancies, disease duration, the presence of erosions, rheumatoid factor (RF), and anti-citrullinated protein antibodies (ACPA) are recorded.

### Assessment of disease activity

Disease activity was measured with DAS28(3)CRP at each visit throughout the registry period. The score is based on the count of twenty-eight swollen and tender joints, and C-reactive protein (CRP). This score excludes patient-reported measures, as these are substantially influenced by pregnancy itself, even in healthy women [12, 13]. During the Covid-19 pandemic, many consultations were conducted digitally, and the assessments of tender and swollen joints were reported by the patient. This also applies to some consultations after 2021 (see Table 1). We excluded preconception visits conducted more than 1 year prior to actual conception from the analyses.

**Table 1:** Clinical characteristics

	Summary	n	Missing, n (%)
Total		598	
Age in years, mean (SD)	31.6 (4.4)	598	0
Disease duration in years, mean (SD)	5.7 (4.8)	479	119 (20%)
BMI, mean (SD)	24.9 (5.1)	556	42 (7%)
DAS28–CRP3, mean (SD)	2.34 (0.92)	505	93 (16%)
Erosive disease, n (%)	154 (34%)	457	141 (24%)
ACPA positive, n(%)	351 (64%)	548	50 (8%)
RF positive, n(%)	291 (55%)	529	69 (12%)
RF and/or ACPA positive, n(%)	388 (70%)	552	46 (8%)
Smoked first trimester or when first registered, n(%)	18 (3%)	590	8 (1%)
Childless, n (%)	273 (46%)	598	0
Patient reported joint count, n (%)	328 (10%)	3225	613 (19%)
Year of delivery, n (%)		598	0
Before 2016	106 (18%)		
2016–2019	181 (30%)		
2020–2024	311 (52%)		
Time of first visit, n (%)		598	0
Pre conception	274 (46%)		
1. trimester	219 (37%)		
2. trimester	87 (15%)		
3. trimester	18 (3%)		
Gestational week at enrollment, mean (SD)	13.3 (6.7)	598	0
Disease activity at 1.trimester, n (%)		392	206 (34%)
Remission	278 (71%)		
Low disease activity	44 (11%)		
Moderate disease activity	66 (17%)		
High disease activity	4 (1%)		
Treatment during pregnancy, n (%)		598	0
No medication	180 (30%)		
bDMARD monotherapy	108 (18%)		
csDMARD monotherapy	97 (16%)		
bDMARD + prednisolone	37 (6%)		
csDMARD + prednisolone	26 (4%)		
bDMARD + csDMARD	66 (11%)		
bDMARD + csDMARD + prednisolone	29 (5%)		
Only prednisolone	55 (9%)		

BMI, Body Mass Index; ACPA, anti-cyclic citrullinated protein antibody; RF, rheumatoid factor; DAS28(3)CRP, Disease Activity Score with 28 joints with C-reactive Protein; bDMARD, biologic disease-modifying anti-rheumatic drug; csDMARD, conventional synthetic disease-modifying anti-rheumatic drug

Disease activity levels were categorized according to DAS28(3)CRP: remission (<2.6), low disease activity (LDA) (≥2.6 but ≤3.2), moderate disease activity (MDA) (>3.2 but ≤5.1) and high disease activity (HDA) (>5.1) [14].

### Health-related quality of life (HRQol)

Patient-reported outcome measures (PROMs) were collected at each visit. RAND-36 was used until 2016 when it was replaced by RAND-12. Both are questionnaires covering eight HRQol domains on a 0–100 scale, where higher scores indicate better HRQol [15]. We calculated unweighted composite scores (Physical Composite Summary (PCS) and Mental Composite Summary (MCS)) for both tools as described by Andersen et al. [16]. Modified Health Assessment Questionnaire (mHAQ) assesses the patient’s capacity to perform activities of daily life, with scores ranging from 0 to 3 [17, 18]. Higher scores indicate higher problems with daily tasks. From 2020, mHAQ was no longer routinely recorded in RevNatus at the trimester visits.

### Variables

The analysis method of ACPA and RF differs between hospitals and results are recorded as positive according to local guidelines. Before 2016, these variables were recorded numerically in the register and considered positive for ACPA when > 10 U/ml, and for RF when > 75 IU/ml. To calculate DAS28(3)CRP, CRP was set as 3 in the formula when reported as <5.

### Definition of flare and improvement

The definition of flare and improvement has varied in earlier research [13]. The most common definition of flare is the reversed EULAR criteria, first applied by de Man et al. in 2008 [6, 19]. This defines flare as a change in DAS28(3)CRP > 0.6 if final DAS28(3)CRP > 3.2, or >1.2 at any final score. The EULAR response criteria are commonly used to define improvement [20]. Good or moderate response is defined as a change of >0.6 to <1.2 if the final DAS28(3)CRP < 5.1, or a difference of >1.2 at any level. In our material many pregnancies had lower DAS28(3)CRP levels than 3.2 at all points. Applying the EULAR criteria would have resulted in a skewed comparison of the proportions improving and worsening. For instance, a decrease of 1.0 in DAS28(3)CRP-score would be regarded as improvement, but an increase of 1.0 would not be counted as a flare. We have therefore considered a change of 1.2 as the definition of clinical significant change, both for amelioration and flare [19]. However, to compare our results with earlier studies, we also calculated change in disease activity with the EULAR definitions.

### Medication use

The method of registration of medication in RevNatus has changed over the years. Initially, medications were registered at every visit, but from 2016, the medication was recorded when starting and stopping a new medication. From 2022, information concerning medications during pregnancy was recorded at the first visit after delivery. Medication use before pregnancy was recorded at baseline as a historical binary variable (yes or no). Where available, the exact date of medication discontinuation was documented. Prepregnancy use in this study was defined as any use the last two years preceding pregnancy.

### Statistical analysis

We used a linear mixed model with DAS28(3)CRP as dependent variable and time as a categorical variable with seven categories. The model was with random intercepts and three-leveled, with time point nested within pregnancies and pregnancies nested within women.

The actual time point for the six-week visit varies between 6 weeks and up to 12 weeks. As a sensitivity analysis, we used an eight-category time variable, where the time for the first control postpartum was set to 6 weeks or 12 weeks, whatever closest to the actual time point.

The analysis was repeated with use of TNFi as a time-varying factor, excluding the preconception visit because of the difference in registration of medication pre pregnancy.

To examine if disease activity when entering the pregnancy affected the curve, disease activity at the first trimester and its interaction with time were included as covariates. In this analysis disease activity was dichotomized with DAS28(3)CRP > 3.2 indicating active disease.

To study the presence of RF, ACPA, or erosive disease we added these separately as covariates with its interaction with time. Since ACPA is a plausible confounder for the effect of erosive disease on disease activity, we also added ACPA and its interaction with time in the last analysis.

Time periods were studied by including the year of delivery in three periods and its interaction with time as covariates. The year of delivery was divided into pre-2016, 2016–2019, and 2020–2024. This stratification was based on the timing of publication of EULAR and ACR guidelines.

To study the pattern of mHAQ, Mental Composite Summary score, or Physical Composite Summary score, we used a linear mixed model with one of these as dependent variable and time as a categorical variable.

Normality of residuals was checked by visual inspection of QQ-plots. There were some deviations from normality, so robust variance estimators were used. Analyses with ln(DAS28(3)CRP) were done as sensitivity analyses, data not shown.

Proportions were compared using the Pearson chi squared test. We report 95% confidence intervals (CI) where relevant and considered a two-sided *p* < 0.05 statistically significant. For statistical analysis, we used Stata version 19.5 (StataCorp LLC, College Station, TX, USA).

### Patient involvement

All patients give written consent when joining the register, including later participation in research. Patient representatives participated in the development of the RevNatus registry and in the associated projects based on data from RevNatus.

## Results

### Patient inclusion data/patient recruitment

Between January 2007 and December 2024, 869 women with RA were included in RevNatus. We included a total of 598 births in 475 women in the current analysis, as illustrated in Fig. 1. Eighteen were twin pregnancies.

Not all women had data from every time point. The median number of visits was five (mean 4.4, SD 1.3). As depicted in Fig. 1, some visits had missing DAS28(3)CRP. We excluded postpartum visits occurring more than 15 months after delivery or during a subsequent pregnancy. 136 women (23%) had data from all seven time-points.

### Demographics and disease characteristics

The mean age of the included women was 31.6 years, and the mean disease duration 5.7 years. Table 1 shows the social and demographic characteristics at baseline.

### Disease activity

**Fig. 2:**
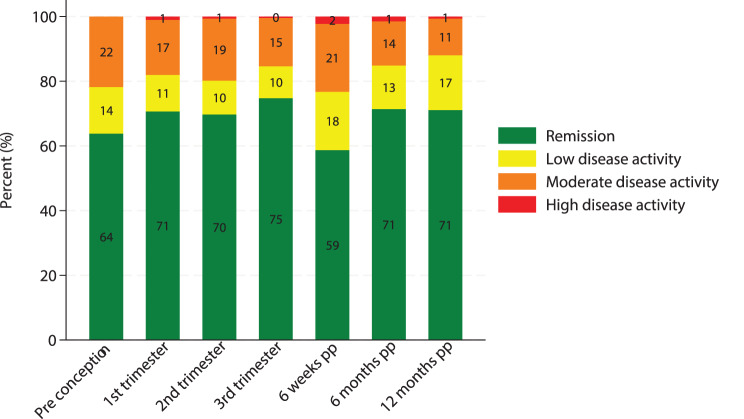
Percentage of women in each EULAR-disease activity category before, during and after pregnancy LDA, low disease activity; MDA, moderate disease activity; HDA, high disease activity; pp, postpartum

Before pregnancy 64% of the women were in remission with a low median DAS28 (3)CRP at 2.04 (mean 2.32, SD 0.91). The fluctuations in activity categories are shown in Fig. 2.

**Fig. 3:**
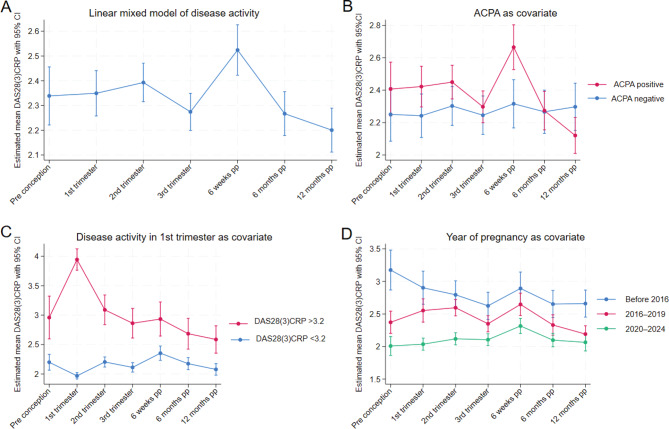
**A-D**: estimated marginal mean Das28(3)CRP (**A**) Total; (**B**) with ACPA as covariate; (**C**) with disease activity in 1st trimester as covariate; (**D**) with year of pregnancy as covariate. ACPA, anti-cyclic citrullinated protein antibody; pp, postpartum; DAS28(3)CRP, disease activity score with 28 joints with C-reactive protein

**Fig. 4:**
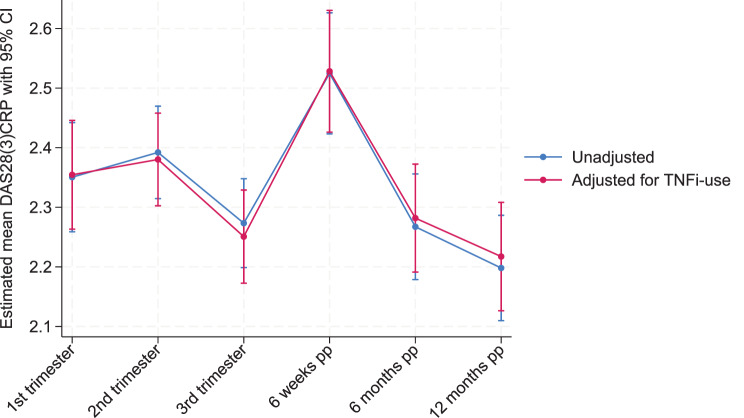
Estimated DAS28(3)CRP score both unadjusted and when adding TNFi-use as time varying factor. Pp, postpartum; TNFi, Tumor necrosis factor inhibitors; DAS28(3)CRP, disease activity score with 28 joints with C-reactive protein

In the first trimester the estimated mean DAS28 (3)CRP was 2.35 (CI 2.26 to 2.44), no significant change from preconception (*p* = 0.86). We observed a small but statistically significant decline in DAS28(3)CRP from the second to the third trimester (from 2.39 to 2.27, *p* = 0.003). From the third trimester to the six-week postpartum visit, DAS28(3)CRP increased to 2.52 (*p* < 0.001). The sensitivity analysis showed that this increased level was also present in week 12 (2.48, *p* = 0.013). The activity then fell to preconception levels at six months (2.27, CI 2.18 to 2.36) and at one year postpartum (2.20, CI 2.11 to 2.29). The curve is displayed in Fig. 3A. Adding use of TNFi as a time-varying factor did not yield any significant differences, se Fig. 4.

Because of the difference in gestational age at enrollment, a sensitivity analysis was performed, investigating only those enrolled before pregnancy or in the first trimester. The analysis yielded similar results (shown in supplements, Figure X1).

From the first to the third trimester, 11% of pregnancies had a clinically significant improvement, see Table 2. From the third trimester to the six weeks visit 16% of the women experienced a clinically significant flare.

**Table 2:** Proportions of patients with clinically significant changes in disease activity

		Change in DAS28(3)CRP > 1.2		EULAR criteria	
	Total (n)	Improvement % (n)	Flare % (n)	Improvement % (n)	Flare (%) n
From 1^st^ to 3^rd^ trimester					
All patients	306	11% (33)	7% (21)	23% (71)	10% (31)
Remission	214	0% (0)	9% (20)	6% (13)	11% (23)
Low disease activity	39	18% (7)	3% (1)	54% (21)	18% (7)
Moderate/high disease activity	53	49% (26)	0%(0)	70% (37)	2% (1)
ACPA positive	175	14% (25)	7%(12)	25% (44)	9% (16)
ACPA negative	107	6% (6)	7% (8)	21% (22)	13% (14)
From 3^rd^ trimester to 6 weeks postpartum					
All patients	358	6% (22)	16% (58)	15% (55)	20% (71)
Remission	178	2% (4)	13% (24)	9% (16)	17% (30)
Low disease activity	33	15% (5)	24% (8)	30% (10)	27% (9)
Moderate/high disease activity	47	11% (5)	15% (7)	36% (17)	21% (10)
ACPA positive	209	5% (10)	19% (39)	14% (29)	23% (48)
ACPA negative	120	8% (10)	11% (13)	19% (23)	13%(16)

Stratified by ACPA-status and disease status in first trimester

ACPA, anti-cyclic citrullinated protein antibody; Das28(3)CRP, Disease activity score with C-reactive protein; EULAR, The European Alliance of Associations for Rheumatology

The patterns of disease activity differed between participants with high versus low disease activity in the first trimester (Fig. 3C). The active disease group experienced a significant decline to third trimester (−1.08, *p* < 0.001), but no significant flare postpartum. Those in remission experienced a small, but statistically significant increase in DAS28(3)CRP from 1.97 1^st^ trimester to 2.11 in the 3^rd^ trimester (*p* < 0.001). For comparison, we repeated the analysis in women in remission without the history of biologics. We found no significant increase in disease activity from 1st to 3rd trimester (shown in supplemental material, Figure X2). Rates of amelioration and flares were different dependent on disease activity in the first trimester; the results are listed in Table 2.

### The effect of antibodies and erosive disease

The patterns of disease activity differed between participants with negative and positive antibodies, see Figure 3B and supplemental Figure X3. Women with positive ACPA had higher disease activity at six weeks postpartum (*p* = 0.001). Patients seronegative for ACPA had stable values during pregnancy and only a minor increase in DAS28(3)CRP-scores at week six that was not statistically significant. We observed the same pattern when analyzing women with and without positive RF, regardless of ACPA-status (Figure X3).

The proportions of flare and improvement are listed in Table 2. For ACPA positive women with DAS28(3)CRP > 3.2 in 1^st^ trimester, 66% (21/32) improved during pregnancy, compared to 22% (4/18) in the seronegative group (*p* = 0.003). The difference in flare rates postpartum dependent on ACPA-positivity was not statistically significant (*p* = 0.061).

Erosive disease did not affect DAS28(3)CRP scores during pregnancy, but at six weeks postpartum, the score was higher (2.47 vs 2.76, *p* = 0.024). When adjusting for ACPA, the difference was no longer significant, see Figure X4 in supplements.

### Variations in time periods

When comparing the different time periods, we found different patterns of disease activity depending on the year of the pregnancy, see Fig. 3D. Pregnancies before 2016 had a significant decline in mean DAS28(3)CRP from 1^st^ to 3^rd^ trimester (from 2.90 to 2.63, *p* = 0.016), while the more recent pregnancies did not experience this decrease. Even though the 2020–2024 group had low activity preconception and during pregnancy, there was a significant increased activity at six weeks postpartum (from 2.11 to 2.32, *p* < 0.001). The pregnancies after 2020 had lower DAS28(3)CRP at all registrations in pregnancy and 6 weeks postpartum compared to the other groups. When examining postpartum flares at six weeks, the percentages from the earliest to the latest period were declining; 23%, 17%, and 12%.

### PROMS

**Fig. 5:**
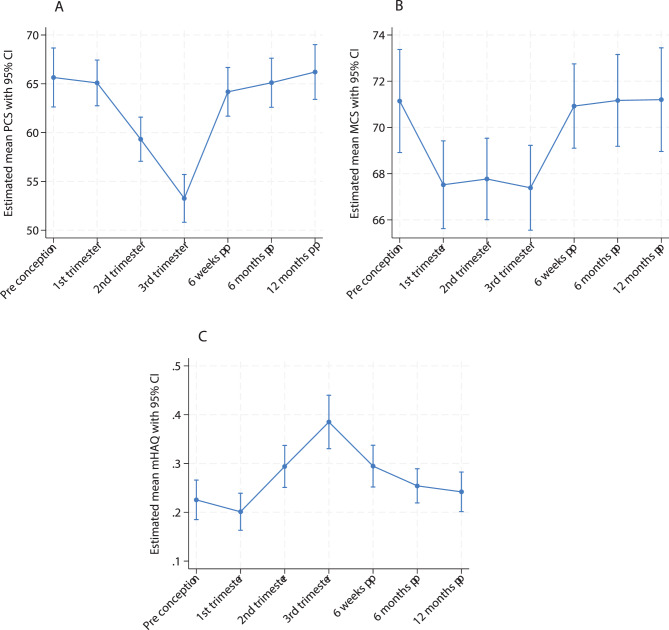
**A-C** Estimated marginal mean scores of (**A**) Physical Composite Summary score (PCS), (**B**) Mental Composite Summary score (MCS), (**C**) Modified Health Assessment Questionnaire (mHAQ)

The changes in mHAQ, MCS score and PCS Score are shown in Fig. 5A–C. All three scores indicated lower HRQol or function in pregnancy. MHAQ- and PCS-scores were significantly worse in the third trimester, before improving after birth. The median mHAQ in 3^rd^ trimester was 0.25 (IQR 0 to 0.63), and 0.12 (IQR 0 to 0.38) at six weeks.

### Use of medication before, during, and after pregnancy

The percentage of women using medication before, during, and after pregnancy, and the change over the years are described in Table 3.

**Table 3:** Use of medication before, during and after pregnancy

	Used before pregnancy		Used in 1^st^ trimester	Used in 2^nd^ trimester	Used in 3^rd^ trimester	Used 6 weeks pp	Used 6 months pp	Used 12 months pp
TNFi								
All, n/N (%)	285/564 (51%)		230/576 (40%)	139/588 (24%)	68/588 (12%)	220/595 (37%)	254/510 (50%)	183/357 (51%)
Before 2016, n/N (%)	44/101 (44%)		18/89 (20%)	1/96 (1%)	1/97 (1%)	26/105 (25%)	31/98 (32%)	25/78 (32%)
2016–2019, n/N (%)	71/172 (41%)		54/176 (31%)	26/181 (14%)	15/180 (8%)	65/180 (36%)	79/161 (49%)	69/129 (54%)
2020–2024, n/N (%)	170/291 (58%)		158/311 (51%)	112/311 (36%)	52/311 (17%)	129/310 (42%)	144/251 (57%)	89/150 (59%)
Missing, n	34		22	10	10	3	88	241
Other b/tsDMARDs								
All, n/N (%)	25/547 (5%)		10/575 (2%)	8/586 (1%)	4/585 (0.7%)	11/594 (2%)	15/510 (3%)	13/357 (4%)
Before 2016, n/N (%)	1/101 (1%)		1/88 (1%)	0	0	1/105 (1%)	3/98 (3%)	3/78 (4%)
2016–2019, n/N (%)	7/171(4%)		1/176 (0.6%)	1/180 (0.6%)	0	3/180 (2%)	4/161 (3%)	5/129 (4%)
2020–2024, n/N (%)	17/275 (6%)		8/311 (3%)	7/304 (2%)	4/311 (1%)	7/309 (2%)	8/251 (3%)	5/150 (3%)
Missing, n	51		23	12	13	4	88	241
Metotrexate								
All, n/N (%)	379/551 (69%)		2/573 (0.3%)	1/584 (0.2%)	0/582	26/593 (4%)	67/502 (13%)	69/397 (19%)
Before 2016, n/N (%)	37/65 (57%)		1/89 (1%)	1/95 (1%)	0	4/105 (4%)	15/98 (15%)	25/78 (32%)
2016–2019, n/N (%)	127/176 (72%)		1/174 (0.6%)	0	0	17/180 (9%)	31/161 (19%)	26/129 (20%)
2020–2024, n/N (%)	215/311 (69%)		0/309 (0%)	0	0	5/309(2%)	21/251 (8%)	18/150 (12%)
Missing, n	47		25	14	16	5	96	201
Sulfasalazine								
All, n/N (%)	NA		160/577 (28%)	155/586 (27%)	152/585 (26%)	121/594 (20%)	116/502 (23%)	89/397 (25%)
Before 2016, n/N (%)	NA		25/90 (28%)	22/94 (23%)	25/94 (27%)	24/105 (23%)	26/78 (27%)	23/78 (30%)
2016–2019, n/N (%)	NA		46/176 (26%)	48/181 (27%)	47/180 (26%)	45/180 (25%)	40/161 (25%)	31/129 (24%)
2020–2024, n/N (%)	NA		89/311 (29%)	85/311 (27%)	80/311 (26%)	52/309 (17%)	50/251 (20%)	35/150 (23%)
Missing, n	NA		21	12	13	4	96	201
Prednisolone used during pregnancy								
All, n/N (%)		147/567 (26%)						
Before 2016, n/N (%)		58/101 (57%)						
2016–2019, n/N (%)		39/173 (23%)						
2020–2024, n/N (%)		50/293 (17%)						
Missing, n		31/598						

Other b/tsDMARDS includes rituximab, abatacept, tocilizumab, JAK-inhibitors. TNFi, Tumor necrosis factor inhibitors; pp, postpartum; b/tsDMARDs:, biological and targeted synthetic disease-modifying anti-rheumatic drugs; NA, Not Available

A total of 51% had a history of TNFi use before pregnancy, and 40% had at least one injection or infusion during the first trimester. The proportion of women using medication declined in the third trimester, and increasing to 12 months postpartum, where over half of the patients were on biologics.

Methotrexate is a known teratogen and stopped when planning pregnancy. In our material, two women used methotrexate in early pregnancy because of unplanned pregnancies. Both reported healthy newborns. Sulfasalazine was used at a stable percentage of around 27% during pregnancy. Preconception use was inconsistently reported and excluded.

The percentages of women using biologics during pregnancy changed remarkably from 19% (20/106) before 2016 to 30% (55/181) in 2016–2019 and 53% (165/311) in the latest period (2020–2024). The percentage of women using sulfasalazine was stable over the years.

Since prednisolone is often tapered gradually, the correct dose at the different time points were uncertain and not reported. Twenty-six percent of the women used prednisolone at one time during pregnancy. When examining prednisolone use over time, the percentages from the earliest to the latest period were declining; 57%, 23%, and 17%.

## Discussion

Disease activity in women with RA from preconception to one year postpartum showed an overall trend with low stable disease through the pregnancy, the lowest disease activity in the third trimester, but a significant flare in the first three months after delivery. At one year postpartum, activity had returned to prepregnancy levels. Eleven percent improved during pregnancy, and 16% suffered a flare during the first three months postpartum.

However, as our study covered a long period with substantial changes in treatment, the interpretation of how the disease varied during pregnancy needs to be nuanced. Both the time trend and the subgroup analyses showed significant differences in how the activity varied throughout pregnancy, depending on the disease activity at the beginning of the pregnancy. This is consistent with earlier findings [5, 6, 21]. When considering those with moderate or high activity in first trimester, our data show amelioration in 49% (70% with the EULAR definition), which is closer to the 60% described by Jethwa et al. [2] Preconception disease activity was higher in many of the included studies in the metanalysis; in the largest cohort, only about 10% had DAS28(3)CRP < 2.6 [6]. This illustrates the difficulty of comparing flare and improvement rates across studies and highlights the need to account for baseline disease activity.

When we added TNFi-use as a time-varying factor, the results did not change significantly. This observation is, however, confounded by indication, since those with high disease activity are more likely to also continue or start treatment.

Most women in our cohort were in remission, with low baseline DAS28(3)CRP. In this setting, the question of potential amelioration is less important than the risk of getting worse, especially if considering stopping medication. For women in remission in the first trimester, we observed a slight increase in disease activity during pregnancy and a more pronounced increase at six weeks. Since many women stop medication in first trimester because of low disease activity, one may speculate if the increase reflects the result of this decision. When performing the same analysis in women in remission without any history of bDMARDs, there was no increase from 1^st^ to 3^rd^ trimester (Figure X2 in supplements). This gives support to the hypothesis, but the question needs to be further explored.

In our study 16% experienced postpartum flares which is lower than in most previous studies (30%-92%) [2, 6, 22–27], including those with low baseline disease activity [25]. Our definition is more conservative than the reversed EULAR criteria, but even when applying the EULAR criteria, the proportion is lower (20%). A study by Smeele et al. from 2021 [3] did not find any postpartum flares. In that study, women were treated with a tight treat-to-target regimen throughout pregnancy, which may have prevented flares. It is therefore not directly comparable with our register-based study. It does, however, highlight the importance of clinical monitoring during and after pregnancy to identify worsening in disease activity. The systematic followup in our cohort likely contributed to the observed low flare rate, in addition to the low baseline disease activity. Since 2020, the standard approach in Norway has been to resume treatment shortly after delivery, a strategy that may have contributed to reducing the risk of flare. Whether treatment with bDMARDs in the late stages of pregnancy has an additional positive effect against postpartum flare is still unclear.

Another interesting finding is that the observed fluctuations in disease activity during pregnancy and postpartum seem to be most present in the ACPA-positive women, and with no significant changes in DAS28(3)CRP in the seronegative group. This both supports and contradicts earlier findings. Some previous studies found higher disease activity in those with positive ACPA [5, 23, 28], but two other studies did not find any difference [4, 6]. One of the studies analyzed the risk of flare, not changes in disease activity [4]. Even though our method was similar to de Man et al. [6] and the percentage of ACPA-positive patients is comparable, the Dutch study included fewer patients and the mean DAS28(3)CRP was considerably higher. Our material is larger, increasing the power to discover differences.

Another Dutch study from the same cohort reported more frequent amelioration in seronegative patients [29]. Our findings directly contradict this, both when analyzing all pregnancies and only those with DAS28(3)-CRP > 3.2 in 1^st^ trimester. In the PARA-cohort, 75% improved among the seronegative, and 39% of the seropositive, as opposed to in our material with 22% and 66%, respectively. Our findings align with the other results in our data where ACPA-positive women had higher disease activity, and pregnancies with higher activity had higher rates of improvement. Although the reasons for this difference from the earlier study are unclear, the findings nevertheless support the same conclusion drawn by the authors that RA is a heterogeneous disease involving different pathogenic mechanisms [29].

Erosive disease did not have any effect on predicting disease activity, and the presence of erosive disease is declining. Though presence of erosive disease is a marker for more aggressive disease, the actual disease activity and serological status are more important to consider when advising patients planning pregnancies.

Earlier studies have shown misalignments when comparing patient-reported measures against DAS28(3)CRP [12, 13]. In our study MCS, PCS and mHAQ reflected worse HRQol and physical function at times when DAS28(3)CRP indicated low disease activity. It is interesting to note that the scores in this cohort were lower than reported in earlier studies, with median mHAQ score third trimester being 0.25 compared to 0.9–1 reported in previous studies [12, 24]. This supports that the use of mHAQ in evaluation of disease activity is less relevant now than in earlier cohorts and that mHAQ may misrepresent disease activity in pregnancy.

When looking at how the progression of disease activity differs across years, we found that the latest pregnancies had the lowest DAS28(3)CRP at all registrations. We also found that the fluctuations of disease activity were different in pregnancies before 2016 compared with the group after 2020. Some of the differences in improvement and flare rates across earlier studies have been attributed to variations in methodology, disease assessment, and prospective vs. retrospective designs. These points are still valid, but our study shows that even when these factors were the same, the curve differed depending on the period of pregnancy.

The effect of medications on disease activity in pregnancy is hard to study in observational studies because the disease activity itself affects whether the woman receives any treatment at the times of registration. We do not think that other factors can explain the difference in trends. We therefore claim that these time trends can be a different way of visualizing the effect of treatment, by showing how disease progression has changed in line with developing guidelines. This can indicate that a more liberal use of bDMARDs has led to lower disease activity both before, during, and after pregnancy. This is an important point to consider when comparing studies and advising our patients, since the women we see pregnant today do not necessarily experience the same disease course as pregnant women 10 years ago.

In earlier studies the pregnant women were largely untreated, and the fluctuations could therefore be interpreted as an effect of the pregnancy itself. In our cohort about 70% use medication at some point during pregnancy, so this assumption is no longer the case. Our postulate is that the ameliorating effect of the pregnancy can be relevant for women with high disease activity without medication but should not be an alternative to medical treatment. The effect of bDMARDs seems to be more pronounced than the ameliorating effect of pregnancy itself.

When considering treatment in pregnancy, the benefit of treatment needs to be balanced against the potential risk for the fetus. We know that high disease activity during pregnancy gives a higher risk of adverse pregnancy outcomes [30]. Previous studies have not found an increased risk of malformations, miscarriage or other adverse pregnancy outcomes with TNFi use in pregnancy [31]. Another potential risk is infection, both in the mother and the newborn. Pregnant women in general have a higher risk of infection than non-pregnant women, and although the data is limited, some studies suggest that this risk is slightly increased in women using TNFi in pregnancy [32]. It is not known if the length of treatment or type of TNFi affects the risk. Earlier studies have shown conflicting results regarding the risk of infections for children exposed to TNFi in utero [32]. Exposed children are advised against live vaccines their first year, however new guidelines recommend the administration of Rotavirus vaccine [31, 33]. Although the association between high disease activity during pregnancy and outcomes is well established, data on the associated risks for the offspring remain limited, highlighting an important gap for future research.

Our study has several strengths, most importantly the size, the prospective design, and the statistical method. It is to our knowledge the largest study to date investigating these aims, making it possible to analyze subgroups. It also has a long time span, encompassing women with and without bDMARDs. RevNatus is the oldest register of its kind, and because of the way health services are organized in Norway, most pregnant women with RA are eligible for inclusion, not dependent on social, economic, or geographical factors. Recruitment takes place in rheumatology departments at university hospitals, regional/community hospitals, and private practices, making the risk for selection bias low.

There are some limitations to our study. Not all women had all seven registrations, and missing data is therefore an issue. One would suspect that women with low disease activity have a reduced need to attend clinical visits, giving the risk of overestimating disease activity. The baseline activity is, however, low, and an overestimation would strengthen our findings with increased activity at the six-week visit.

Most Norwegian women with RA are followed in rheumatology departments, and the majority of women planning a pregnancy or become pregnant are included in RevNatus. Some patients with low disease activity for a longer period and without the need for medication may be followed in primary care, and these results may not be generalizable to these women. The cohort consists predominately of white women and may not be generalizable to patients with other ethnicities.

Ten percent of the registered visits were conducted digitally, with patient reported joint counts. A large meta-analysis from 2021 by Rampes et al. [34], found that patient-reported joint counts had good correlation with clinician-reported counts for low disease activity. The mean DAS28(3)CRP for patient-reported visits was low, 1.96 (SD 0.49), and we think this has not had any significant influence on our results.

The registration of the use of medication will never be as precise in a register like RevNatus compared to RCTs. Due to the way use of medication was registered during the period 2016–2021, we cannot tell if some of the women were not using medication or if data were missing. Thus, we may have underestimated the use of medications in some patients, but with no likely significant impact on the results.

## Conclusion

The present study indicates that disease activity during pregnancy in women with RA depends on disease activity preconception and proven seropositive status. Most women had low and stable disease activity during pregnancy, but postpartum flares still occurred. Time trends indicate that the increased use of bDMARDs in recent years, may have led to a higher proportion of women in remission through pregnancy and fewer flares postpartum.

## Electronic supplementary material

Below is the link to the electronic supplementary material.

Supplementary material 1

## Data Availability

The data that support the findings of this study are available from the RevNatus registry. Restrictions apply to the availability of these data, which were used under license for the current study, and are not publicly available. The data cannot be shared publicly due to the requirements of the register holders involved and the national general data protection regulation, to protect the privacy of individuals. A de-identified patient data set can be made available to researchers upon reasonable request. The data will only be made available after submission of a Project plan outlining the reason for the request and any proposed analyses and will have to be approved by the RevNatus steering group. Project proposals can be submitted to the corresponding author ingusagb@stud.ntnu.no. Data sharing will have to follow appropriate regulations including GDPR.

## Abbreviations

ACPA: Anti-cyclic citrullinated protein antibodies
ACR: The American College of Rheumatology
bDMARDs: Biological disease-modifying anti-rheumatic drugs
CRP: C-reactive protein
csDMARD: Conventional synthetic disease-modifying anti-rheumatic drug
DAS28(3)CRP: Disease Activity Score in 28 joints with C-Reactive Protein
EULAR: The European Alliance of Associations for Rheumatology
HDA: High disease activity
HRQol: Health-related quality of life
LDA: Low disease activity
MCS: Mental Composite Summary
MDA: Moderate disease activity
mHAQ: Modified Health Assessment Questionnaire
NKSR: The Norwegian National Network for Pregnancy and Rheumatic Diseases
PCS: Physical Composite Summary
pp: postpartum
PROMs: Patient-reported outcome measures
RA: Rheumatoid arthritis
RF: Rheumatoid factor
TNFi: Tumor Necrosis Factorα Inhibitors
