# Supplementary material for: The biologic era and pregnancy in rheumatoid arthritis: a prospective multicenter study from the Norwegian RevNatus registry
Source: BMC Rheumatol. 2026 Jun 3;10:63. doi: 10.1186/s41927-026-00663-7 (PMC13449393; doi:10.1186/s41927-026-00663-7)
Supplement: Supplementary file 1 [file 41927_2026_663_MOESM1_ESM.docx]

Supplemental files

Figure X1 – Estimated marginal mean disease activity in women enrolled before pregnancy or in 1^st^ trimester, showing similar results to the overall cohort. Pp, postpartum; DAS28(3)CRP, Disease activity score 28 joints with C-reactive Protein.

Figure X2 – Estimated marginal mean disease activity in 1^st^ trimester as covariate showing s slight increasing trend in the low activity group in 3A, but no change from 1^st^ to 3^rd^ trimester in 3B. A – Total cohort. B – Only women with a history of bDMARDs use. *DAS28(3)CRP in 1^st^ trimester. Pp, postpartum; DAS28(3)CRP, Disease activity score 28 joints with C-reactive Protein. bDMARDs, biological disease-modifying anti-rheumatic drugs.

Figure X 3 – Estimated marginal mean DAS28(3)CRP with rheumatoid factor (RF) as covariate. Pp, postpartum; DAS28(3)CRP, Disease activity score 28 joints with C-reactive Protein.

Figure X 4 – Estimated marginal mean with erosive disease as covariate, adjusted for ACPA. Pp, postpartum; ACPA, Anti-citrullinated protein antibody; DAS28(3)CRP, Disease activity score 28 joints with C-reactive Protein.
